# Data Security and Trading Framework for Smart Grids in Neighborhood Area Networks

**DOI:** 10.3390/s20051337

**Published:** 2020-02-29

**Authors:** Jayme Milanezi Junior, João Paulo C. L. da Costa, Caio C. R. Garcez, Robson de Oliveira Albuquerque, Arnaldo Arancibia, Lothar Weichenberger, Fábio Lucio Lopes de Mendonça, Giovanni del Galdo, Rafael T. de Sousa

**Affiliations:** 1 Department of Electrical Engineering, University of Brasília, Brasília 70910-900, Brazil; joaopaulo.dacosta@ene.unb.br (J.P.C.L.d.C.); caio.garcez@redes.unb.br (C.C.R.G.); robson@redes.unb.br (R.d.O.A.); fabio.mendonca@redes.unb.br (F.L.L.d.M.); desousa@unb.br (R.T.d.S.J.); 2 Brazilian Electricity Regulatory Agency, ANEEL, Brasília 70830-110, Brazil; 3 Elektronische Fahrwerksysteme GmbH, 85080 Ingolstadt, Germany; arnaldo.arancibia@efs-auto.de (A.A.); lothar.weichenberger@efs-auto.de (L.W.); 4 Institute for Information Technology, Ilmenau University of Technology, 98693 Ilmenau, Germany; giovanni.delgaldo@iis.fraunhofer.de; 5 Fraunhofer Institute for Integrated Circuits IIS, 98693 Ilmenau, Germany

**Keywords:** smart grid privacy, energy trade, traffic analysis attack, cryptography

## Abstract

Due to the drastic increase of electricity prosumers, i.e., energy consumers that are also producers, smart grids have become a key solution for electricity infrastructure. In smart grids, one of the most crucial requirements is the privacy of the final users. The vast majority of the literature addresses the privacy issue by providing ways of hiding user’s electricity consumption. However, open issues in the literature related to the privacy of the electricity producers still remain. In this paper, we propose a framework that preserves the secrecy of prosumers’ identities and provides protection against the traffic analysis attack in a competitive market for energy trade in a Neighborhood Area Network (NAN). In addition, the amount of bidders and of successful bids are hidden from malicious attackers by our framework. Due to the need for small data throughput for the bidders, the communication links of our framework are based on a proprietary communication system. Still, in terms of data security, we adopt the Advanced Encryption Standard (AES) 128 bit with Exclusive-OR (XOR) keys due to their reduced computational complexity, allowing fast processing. Our framework outperforms the state-of-the-art solutions in terms of privacy protection and trading flexibility in a prosumer-to-prosumer design.

## 1. Introduction

The power grid is a crucial large-scale infrastructure. In order to allow high levels of automation, information security, distributed energy control, and robust load fluctuation management of the power grids, smart grids (SGs) are essential. Several interdisciplinary aspects are treated in SG such as interoperability, information security, scalability, reliability, energy efficiency, reusability, communication backbone, electrical actuators, and sensor and control technologies [1]. Although the SGs are constantly improved, they are still vulnerable to cyber attacks. Hence, current power grids should be further improved to fit the demands regarding data security [2] and energy trade between prosumers [3,4].

To the best of our knowledge, there are open issues in the literature with respect to data protection associated with trade prices between producers. For instance, the main efforts towards privacy consists of obfuscating the instantaneous consumption pattern of each consumer [5,6]. This is generally accomplished by hiding the instantaneous power consumption of the client as fine-grained data can reveal in detail the lifestyle of the consumer [7,8]. However, the profile of traded energy also delivers relevant information about prosumers to their neighbors. As in [9], the ability to link the bids to individual consumers allows the untrusted entity to build up a profile of the consumer’s behavior. In particular, the quantities of traded energy can be very informative about the economical welfare of the owner [10]. Privacy requirements dictate that prosumers cannot gain information regarding other prosumers’ consumption and production—not even if they are trade partners [11]. Models dealing with energy trade directly among prosumers [3,12] limit themselves to exploiting the trade environment without discussing in detail data-security aspects related to the identities of the traders in relation to their neighbors. As a consequence, several topics related to privacy requirements are still open in SGs, such as power production and bidding in trading systems.

In this paper, we consider the problem of providing data privacy for self-interested players that trade energy in the context of a Neighborhood Area Network (NAN). The energy is sold by local micro-generators and locally purchased by their neighbors, also known as the final users. Our framework deals simultaneously with SG data-security requirements and energy-trade systems. As a first contribution, the proposed framework has a privacy-preserving model which has a low computational complexity and avoids completely an unauthorized party to identify the bidders, the number or types of them, and even whether the bids achieve a deal. As a second contribution, all the bids are made clear to the NAN participants, with all Smart Meter (SM) owners having access to how many bids are proposed and their types, prices, and quantities. As a third contribution, we propose a clearing price mechanism that allows for each a Smart Meter (SM) to calculate the final prices and respective amounts of energy that are traded, including a financial reward to the power-grid company. Nevertheless, the proposed framework avoids totally any access to the bidders identities. Once each node learns the bids, it processes individually the information obtained in order to know the quantities and prices effectively traded. The communication links are provided by a patent-pending proprietary communication system [13].

The remainder of this paper is as follows. Section 2 surveys the state-of-the-art in terms of SG-security requirements and energy-trading schemes for electricity markets. We use the technical requirements observed in the literature to present a detailed problem description in Section 3. Section 4 details the proposed framework for energy trade in a NAN. Section 5 shows the results, and Section 6 concludes the paper.

## 2. SG-Trading Systems and Data Security—State-of-the-Art

In this section, we provide an overview of the literature with respect to trading systems and data security in SG. First, we present basic characteristics of trading systems for connected prosumers in Section 2.1 and auctions in Section 2.2. In Section 2.3 and Section 2.4, data security towards privacy and cryptographic systems applied to smart grids are revised, respectively. Additionally, in Section 2.5, blockchain-based transactive energy systems are reviewed.

### 2.1. Trading Systems for SG Prosumers

In a Neighborhood Area Network (NAN), each household unit is represented as a Home Area Network (HAN) and is equipped with an SM [14]. Some of the HANs are prosumers that, in some occasions, export energy to the grid from their Distributed Energy Resources (DER).

There are three different compensation mechanisms in [15] associated to topologies of DER installation and billing regimes. The first mechanism is the Net Energy Metering (NEM) that allows a DER that is generating a surplus of electricity to export the excess to the grid, earning a corresponding credit in kilowatt-hours (kWh). In order to correctly effectuate the measurements, in the NEM, the SM is bidirectional. The second scheme is the Buy All, Sell All, an arrangement that provides a standard sell rate to a DER system for all of the electricity they generate. In Buy All, Sell All schemes, the HAN cannot consume the energy that the DER produces, exporting it entirely. The third arrangement is Net Billing, in which the DER system owner can consume electricity generated by the DER in real time and can export any generation in excess of on-site consumption to the grid—all net energy exports are metered and credited at a predetermined sell rate without kWh banking. For a more detailed review of the features of the three compensation mechanisms, we refer to [15].

Since the prosumers in a NAN are able to trade their energy not only with their neighbors but also with the utility company, the aforementioned mechanisms must consider consumer-to-consumer trade designs. The NEM characteristic of not allowing financial reward for the exceeding energy makes it unable to use the proposed frameworks, which are applicable to the Buy All, Sell All and Net Billing systems.

From the standpoint of energy trade, there are two predominant perspectives about the nature of electricity as a commercial good. When energy is seen as a public service, the tendency is the proposal of a cooperative game among non-selfish players, as in [16,17,18], where the underlying goal is market control and the achievement of social fairness. Alternatively, when the main goals are market efficiency and decentralization [19,20,21], energy is seen as commodity to be traded and a free market model is sought. In this paper, we align our analysis to the latter group. Table 1 summarizes the division of approaches in the literature with regards to the commercial treatment of energy.

With regards to energy prices in the SG, the consumers can purchase energy either from the power grid or from other prosumers. In the first case, a key aspect is that the utility company, which is the company responsible for managing all power grid assets and its operation, usually sells the energy for a unitary price $P_{u}$ to its consumers and purchases the energy from the prosumers by a smaller price, $P_{l}$ [15,16,43]. Therefore, an interval of energy unitary prices that enable trades between prosumers and consumers is given by $P_{l}<P_{b}<P_{u}$, where $P_{b}$ is the price of local traders with which all final users obtain profit with regards to $P_{u}$ and $P_{l}$, since every purchaser is supposed to prefer paying $P_{b}<P_{u}$ for the kWh, with a symmetric interpretation by the side of the sellers.

### 2.2. Auctions in the Electricity Systems and the Preston McAfee

Auction mechanisms have been the cornerstone of many applications in wholesale and retail electric power markets [40]. They are recurrent in electricity market, either under competitive or cooperative frameworks [34,36,44], as a way of obtaining prices, especially in the case of competitive trade mechanisms.

An auction is a method of allocating goods with an explicit set of rules determining resource allocation and prices on the basis of bids from the market participants [45]. According to [46], auctions can be single dimensional, when the price is the only factor taken into account, or multidimensional, when other aspects are distinguished, such as product quality. In a one-sided auction, bidders are either purchasers or sellers and the auctioneer is responsible for deciding which is the winning bid, differing from two-sided auctions, in which both sellers and purchasers offer bids. In an open-cry auction, every bidder has access to every other bid, whereas in a sealed-bid auction, only the auctioneer has access to the offers of all bidders. Single-unit and multi-unit auctions refer to when there are one or several units of a given good; they differ from combinatorial auctions, where multiple, heterogeneous goods are auctioned simultaneously.

According to [47], an ideal double-auction mechanism would satisfy the following properties:

- Individual Rationality (IR)—A trading user should have positive utility. The IR is a necessary property for a price mechanism.
- Incentive Compatibility (IC)—Reporting the true value is a dominant strategy.
- Balanced Budget (BB)—The auctioneer should not lose or gain from the trade. For real-world applications, if the auctioneer does not have to subsidize the trade (called weakly BB property), then it is acceptable.
- Economic Efficiency (EE)—The social welfare should be maximized.

In [48], it has been shown that it is impossible for a mechanism to satisfy all of the four properties. Trade-reduction mechanisms and its variations such as the Preston McAfee’s Double Auction Protocol (PMD) [49] are IR, IC, and weakly BB [47]. The McAfee’s PMD is said to be weakly BB [47], i.e., a system in which the auctioneer does not lose but may gain money. A strongly BB system is observed when the auctioneer does not lose nor gain any money [50].

Auctions are frequently analyzed in terms of the social welfare that they provide. According to [10], social welfare is the sum of consumer surplus, given by the difference between willingness-to-pay and clearing price, and producer surplus, which is the difference between clearing price and costs. In [51], an agent competition double-auction mechanism is proposed to simplify decision making and to promote transactions for the customer-to-customer marketplaces. A quasi-linear utility function is assumed for each bidder. Such function is presented as the difference between the valuation of the item and the amount of money that each bidder actually receives or pays for. A comprehensive analysis of literature towards maximization of social welfare and minimization of aggregated power consumption for Demand Response (DR) programs is presented in [52]. Ref. [37] examined the effect of energy storage on the SG in terms of global social welfare, considering that agents have homogeneous efficiency and running costs. The work in [26,53] shows mechanisms for social welfare calculations in a DR environment.

A common aspect in [10,16,19,26,37,52,53,54] is that they do not take into account the welfare of the utility company itself. It is evident that a great part of the social welfare losses in current applications lie with the utility companies, as they are resistant against energy trade from local prosumers in decentralized generation structures. In this sense, an interesting double-auction model is given by [49], which proposes the Preston McAfee’s Double Auction Protocol (PMD). In this auction, the bids of the *m* purchasers $b_{i}$, i∈{1,2,…,m}, are sorted as in

(1) $$b_{1}\geq b_{2}\geq \cdots \geq b_{m},$$

while the bids of the *n* sellers $s_{i}$, i∈{1,2,…,n}, are

(2) $$s_{1}\leq s_{2}\leq \cdots \leq s_{n}.$$

In PMD, it is necessary to determine the number of bids *k* such that $b_{k}\geq s_{k}$ and $b_{k+1}<s_{k+1}$. As a function of the *k* purchasers’ and sellers’ offers, we calculate the price

(3) $$p_{0}=\frac{b_{k+1}+s_{k+1}}{2}.$$

When the *k*th offer satisfies $s_{k}\leq p_{0}\leq b_{k}$, all the *k* purchasers and sellers trade with price $p_{0}$. Otherwise, only the first (k−1) purchasers and sellers trade and every purchaser pays $b_{k}$ while every seller receives $s_{k}$ when the auctioneer is rewarded with

(4) $$U_{0}=\left(k-1\right)\left(b_{k}-s_{k}\right).$$

According to [55], when the first condition holds, the result is a Pareto efficient mechanism, whereas in the second hypothesis, it is not. Note that McAfee’s double auction does not take into account quantities, as they were idealized for oral double auctions in the stock market [49].

Purchasers and sellers have welfare that are expressed by the positive payoff that they achieve by trading a good with an advantage in terms of their valuations. The welfare of the purchasers is the difference between willingness to pay and the clearing price, while the welfare of the sellers is the difference between the clearing price and costs [10]. Considering $f_{i}$ as the welfare of the purchaser *i* and $g_{j}$ as that of the seller *j*, *I* and *J* are the groups of purchasers and sellers and $d_{i,j}$ is the price of the transaction when purchaser *i* achieves a deal with seller *j*. The social welfare maximization problem is defined in [51] as follows:

(5) $$V\left(f,g,d\right)=\mathrm{Maximize}\;\sum_{i}f_{i}x_{i}+\sum_{j}g_{j}y_{j}-\sum_{i,j}d_{i,j}z_{i,j},$$

subject to

$$\sum_{j}z_{i,j}=x_{i},\;\mathrm{for}\;\mathrm{each}\;i\in I,$$

$$\sum_{i}z_{i,j}=y_{j},\;\mathrm{for}\;\mathrm{each}\;j\in J,$$

$$x_{i}\in \left\{0,1\right\},\;\mathrm{for}\;\mathrm{each}\;i\in I,$$

$$y_{j}\in \left\{0,1\right\},\;\mathrm{for}\;\mathrm{each}\;j\in J,$$

$$z_{i,j}\in \left\{0,1\right\},\;\mathrm{for}\;\mathrm{each}\;i\in I,j\in J,$$

where $x_{i}$ and $y_{j}$ denote if purchaser *i* or seller *j*, respectively, enters a transaction while $z_{i,j}$ denotes if purchaser *i* transacts with seller *j*. Hence, social welfare is defined as the sum of all auctioneers’ payoff and each individual agent’s utility.

### 2.3. Data Security and Privacy in the Smart Grid

According to [56], SG-security objectives are availability, integrity, confidentiality, authentication, authorization, and non-repudiation. Although availability is important to provide network access for end users, data integrity and confidentiality are more critical in the Advanced Metering Structure (AMI) network near the final consumers.

Inviolability of consumption data is at the center of discussions in the realm of SG data-secrecy protection. For instance, data from off-the-shelf SM are sufficient to identify TV movies viewed [57] due to unique fluctuations in the brightness of movies influencing the energy consumption of the TV set. In [8], a study about the impact of data granularity on edge-detection methods, which are the common first step in nonintrusive load-monitoring algorithms, shows that devices in which consumption is above 50 W can be detected. Moreover, data protection is specifically difficult due to the low capacity of the SM in terms of data aggregation and data handling [58].

Masking the identity of each user is the dominant strategy in order to provide user privacy, which is achieved by means of the assignment of false Internet Protocol (IP) data to each SM [11,59]. This technique is however sensitive to de-anonymization, which consists of the reidentification of nodes’ identities behind their false IP. According to the probabilistic frameworks of [60], reported consumed energy on a 10-kWh scale can reduce the percent of reidentified SMs to between 10% and 30%. One should note that it may not be applicable in regions where the law requires that energy reporting should be done with kWh accuracy. In [57], 68% of all consumption data can be reidentified as they have found unique combinations of feature values in the energy-consumption data of 122 households. Updating the pseudonym includes revocation of current pseudonyms and registration of new ones, such that, in order to avoid linkage of the two pseudonyms, after revocating the old one, the customer waits a certain period before registering the new pseudonym [59]. This time interval can be used by a malicious observer for leveraging their reidentification capability. Furthermore, even when SM identity is masked, the problem of mutual identification remains, since all devices must know with whom they are communicating [61]. Hence, a list of the IP numbers should be made available to each node, since a basic principle is that consumers have the right to know where their information is being shared [62].

The use of Internet Protocol (IP) and commercial off-the-shelf hardware and software is one of the most serious vulnerabilities of SG [63]. The Internet as part of the Wide Area Network (WAN) is considered undesirable [62]—such integration entails cyber threats since the SG is based on ethernet, Transmission Control Protocol (TCP)/IP, and other operating systems, thus making the grid more susceptible to attacks. Furthermore, the SG imposes much more strict security requirements than the Internet in order to fully achieve efficient and secure information delivery for critical power infrastructures [56]. Hence, in this paper, we assume that the Internet and off-the-shelf protocols such as TCP/IP are not to be integrated to the SG trading infrastructure.

In [64], the authors propose a secure and privacy-friendly local electricity trading and billing in smart grid that does not require an entity playing the role of a TTP. The following premises are adopted by [64], namely, time synchronization of all entities, secure and authentic communication channel and trading platform serving as a honest-but-curious entity. As shown in Section 5, the state of the art is divided into frameworks considering and not considering the TTP. In constrast to [64] and as shown in Section 2.5, the TTP is imposed by the trade framework based on the existence of the utility company as a neutral player.

### 2.4. Cryptographic Solutions for the SG

Cryptography is a central aspect in SG data security. Several devices that embed cryptographic applications execute their routines using symmetric or asymmetric keys. Each of these keys needs different resources, and in practice, both types of encryption are used [65]. In fact, the state-of-the-art presents a broad division in terms of symmetric and asymmetric keys for SG when the application is related to the SM itself. For instance, the homomorphic Paillier cryptosystem, which is based on the Discrete Logarithm Problem (DLP), is a type of asymmetric key and is proposed as the solution for SM in the solutions presented in [66], while other DLP-based algorithms are also proposed for a SM application in [59,67]. However, in [7], they are described as not desirable for SG, which typically has limited resources. The Paillier encryption is also mentioned as not computationally efficient due to its expensive operations [5,62].

Lightweight keys can serve to protect data as long as the key is inaccessible. In [68], a comparison of computational overheads among XOR, Shamir’s Secret Sharing, and homomorphic encryption is presented. If $C_{1}$ is the cost associated to the XOR operation, $C_{2}$ is the cost associated with the Shamir’s Secret Sharing scheme, and $C_{3}$ is that with homomorphic encryption, then $C_{1}<C_{2}\ll C_{3}$. Due to its extremely low overhead, XOR keys are used in AES, Educational Data Encryption Standard (E-DES), and Blowfish Encryptions [69] and utilized also as encryption method as in [6].

### 2.5. Blockchain in the Smart Grid

Blockchains are designed to achieve peer-to-peer electronic payments directly, without participation of a trusted third party [1] and, as such, they presuppose the lack of a central authority or coordinator from having access to all registers and actions of a network. This assumption collides with the role of the power-utility companies, which are held accountable by the local regulators for the electricity assets in their area, i.e., they are responsible for billing costumers, surveying the use of power grid assets, and further. Moreover, as largely referred to in the state-of-the-art [21,26,33,67,70], a TTP can be adopted for the data-exchange system in the SG. The role of a TTP is frequently assigned to the utility company due to its natural position in the respective SG network. At least in such cases, the financial compensation between traders demands an entity managing the energy exchanges and the respective financial transfers. Hence, due to regulatory and executive aspects, we do not consider blockchains as a feasible solution for NAN centralized trade frameworks.

In [71], a new currency, the NRGcoin, is proposed, however without a detailed description of the compensation mechanism to be mentioned here, i.e., how the financial transfer occurs and who is in charge of surveying it. In [11], a novel blockchain-based transactive energy system is described for energy trade between final prosumers. However, the Distribution System Operator (DSO) is set to ensure the safe operation of the micro-grid and to regulate its total load. In order to achieve the, the DSO can limit the energy and financial assets that the prosumers withdraw for trading. The DSO can also set a price policy for the micro-grid, i.e., the DSO operates as a TTP. For all these limitations, we envisage great challenges to employing blockchains for the specific case of SG trading systems.

## 3. Requirements for the NAN Architecture and Security Framework

The SG characteristics and requirements defined in this section derive from Section 2 and can be referred to as the starting point for the framework to be proposed in Section 4.

As technical requirements for data security in the proposed framework, the number of interactions between a node/unit and the central controller, which is the Trusted Third Party (TTP), as well as between final nodes should be minimal. Each node must have a different AES 128 bit key, and the encrypted messages can be combined with XOR encryption, as its main features are low cost and simplicity. Time stamps must be used as an additional way of ensuring the correctness of the sender identity, constituting an extra argument for symmetric keys. The system must be de-anonymization proof. Neither any attacker nor harmless actors are allowed to know the identities of the bidders, the quantity of them, where there are bids and of which type, or if any deal is achieved. The system must resist traffic analysis attacks. The attacker is supposed to be malicious and powerful, counting on a virtually infinite computational capability.

Although an AES 128 bit encryption key is reputably secure, an active attacker can infer recurrent data and can identify patterns if ciphertexts are repeated. Note that the repetition of ciphertexts in the case of SG trades is likely to occur since prices and quantities of energy can lie around typical values, easing the task of a malicious attacker in identifying the occurrence of offers with similar characteristics. In order to prevent this drawback, we adopt a Linear-Feedback Shift Register (LFSR) [72], which provides a linear function of the previous state of a sequence of bits according to the value of the most left-sited one at each iteration. The initial value of the LFSR is called a seed and the bits that influence the next values of the LFSR are called taps. The period of a LFSR is the minimal number of different outputs before repeating its seed and is given by $p=2^{n}-1$, where *n* is the highest position of the tap that makes the feedback polynomial achieve the maximum possible period. Tables of the taps for maximum-length LFSR in function of each *n* up to 168 bits are given in [73].

In the considered NAN, the aggregator, or central operator, plays the role of a TTP. We use the communication system shown in [13], which provides a reliable wireless intra-battery management system and handles low values of signal-to-noise-plus-interference ratio (SNIR) by varying the length of direct sequences of bits. This is achieved by means of code division multiplexing of several decentralized controllers with a central controller. In doing so, the proposed patent provides a reliable and adaptive link for communication between the TTP, which is the central controller and the consumers in a NAN. The patent in [13] can incorporate different families of codes, including for instance Walsh, Gold, M-sequence, Kasami, and Chaos, as well as different modulation schemes, such as Phase Shift Keying (PSK), Quadrature PSK (QPSK), and Chirp Spread Spectrum (CSS). The system in [13] outperforms systems such as ZigBee, Bluetooth, and LoRa in terms of bit error rate (BER) and latency for critical safety applications.

## 4. Proposed Framework for the NAN Electricity Trading System

The proposed framework is divided into three parts, namely the privacy-oriented data-security system in Section 4.1, the trading system in Section 4.2, and the social welfare of the proposed system in Section 4.3. There are common aspects between the data-security system and the trading system. Therefore, in contrast to the literature, we present a framework taking into account both systems in addition to a clearing price mechanism that includes a financial reward to the power-grid company while keeping all identities secret.

### 4.1. Privacy-Oriented Data-Security System

Since the creation of an AES key between each user and the TTP is a requirement to the proposed framework, a previous step for securely assigning the generated keys is necessary. Therefore, we define the setup zero stage which establishes necessary procedures regarding the purchase, approval, installation, and authentication processes of an SM device to be integrated into the network. The setup zero stage starts by considering that each SM is found accessible for purchase by means of a supplier previously authorized by the power-utility company which is directly linked to TTP in such a way that, during the purchase process, the supplier provides to a platform linked to the TTP, the buyer’s data, as well as a PIN (Personal Identification Number) which allows the TPP to uniquely identify each SM when it performs its first connection to the network.

**Figure 1:**
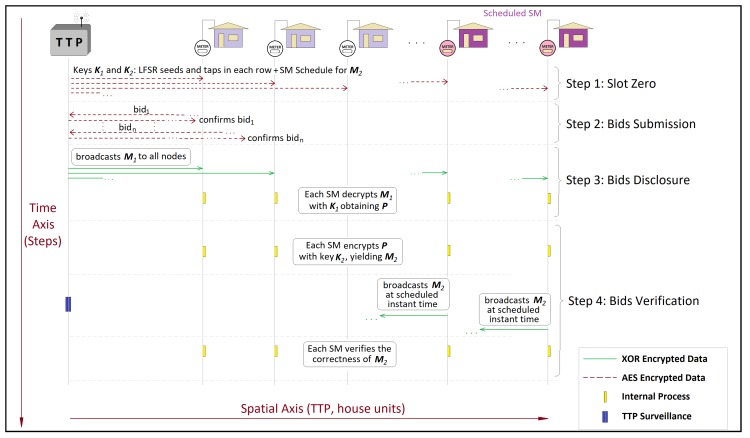
Overall sequence of steps in the proposed framework, with the slot zero and the stages of bids submission, bids disclosure, and bids verification: Note that, only in the slot zero and in the bids submission stage, the ciphertext is obtained via AES. The nodes designed to act as confirmation agents in a given time slot are depicted on the right side of the figure.

After being installed by a TTP-authorized personnel, each SM receives an AES hardcoded key to later perform an authentication process with the TTP regarding the device’s specific keys such as PIN and other data previously assigned by the vendor during the purchase procedure. Once this authentication process is concluded, the privacy-oriented system depicted in Figure 1 takes place.

At this point, it could be argued that employment of asymmetric keys [74] establishes secure communication between the TTP and final nodes. Note that our work intends to offer a low-power processing solution due to the SM-reduced hardware capacity. We intend to avoid key pairs generation. Therefore, we decided to address to the TTP the task of securely assigning to each device a hardcoded AES key. In this sense, the setup zero phase establishes a procedure to be followed by the power-utility company or the local regulator which plays the role of a TTP in our framework.

During a day, regular intervals in which a trading session can happen are called time slots. We adopt 15 min for each slot, as in [11]. The initial time slot is called slot zero, which corresponds to step 1 in Figure 1, when each SM receives from the TTP a ciphertext on AES 128 bit encompassing as contents the XOR keys $K_{1}$ and $K_{2}$ along with the LFSR seeds and taps and the “SM Schedule”. The latter is a list of the time slots in which the respective node must act as a data confirmation agent if a trading session takes place at that time slot. Since 95 slots are specified over a day, the tap of highest order in each LFSR used must be n≥7, in this case, $p=2^{7}-1=127$ different keys. The keys $K_{1}$ and $K_{2}$ are bit matrices with dimensions $K_{1}$ and $K_{2}\in Z^{n\times u}$, where *n* is the number of houses with an SM in the NAN and *u* is the length of each bid. Each row of $K_{1}$ and $K_{2}$ is a different LFSR with its own seed and taps. In this paper, we adopt u=32. Note that the rows $K_{1}$ and $K_{2}$ are updated by the SM itself between two subsequent time slots.

Before a time slot ends, any authenticated node that desires to trade energy forwards to the TTP a purchase or a sell bid, which in Figure 1 occurs in Ssep 2, i.e., the bid submission. The plaintext of the bid, which can only be accessed by the AES key owners, must encompass the bid itself and the IP of the bidder. Only one of the existing AES keys enables the TTP to decrypt this ciphertext successfully, as there is a different AES key per node. The TTP decrypts the ciphertext by using all the existing AES keys until one of them delivers a plaintext that encompasses one of the bidders IP. At this point, the TTP validates the bid if the AES key used to attain the plaintext corresponds to the IP of the respective AES key owner. Please note that, in the considered application, a NAN contains about 100 to 150 house units. We assume that such tests of up to 150 nodes are a reasonable task for the TTP machine. In scenarios where the demand for scalability is necessary, the appropriate procedure is to segment the NAN regions by installing TTP units until computational processing requirements are met. After validating the bid, the TTP uses the same AES key to broadcast a ciphertext of a content that comprises the IP of the bidder, which is the only node able to decrypt this message properly. The bidder thus obtains the confirmation of its order registration. The trading session is open when, at any instant between two time slots, at least one valid bid is decrypted by the TTP.

In every session, a subset *v* of the *n* households forward a bid for purchasing or selling energy such that v≤n. After receiving the *v* offers, the TTP assembles the bit matrix $P\in Z^{n\times u}$, which is the plaintext of all offers. Given that there are only *v* bids, the TTP creates (n−v) false offers and includes all of them in P, observing that the *v* true bids are inserted in random rows. Thus, with the key $K_{1}$, the TTP computes

(6) $$M_{1}=K_{1}⊕P,$$

where ⊕ stands for the XOR operator. Hence, in step 3, the bid disclosure occurs when the TTP broadcasts the cipher matrix $M_{1}$ to all nodes after the end of the time slot in which the bids came up. When the nodes receive $M_{1}$, they learn that a trading session has been created. The SM owners can easily compute P since they have $K_{1}$. They can distinguish the true bids from the false ones as the latter presents inconsistencies in their bit structures, which infringe the rule of bids assemblage, as shown in detail in Section 4.2.

Several problems can affect a trading session based solely on Equation (6), since packet losses, collision, or unfavorable SNIR conditions might prevent some nodes from receiving the cipher matrix $M_{1}$. Therefore, a confirmation step is needed, which is provided by step 4, with bids verification. Each node that receives $M_{1}$ computes a second cipher matrix,

(7) $$M_{2}=K_{2}⊕P,$$

which is the matrix that is used as confirmation data. The matrix $K_{2}\in Z^{n\times u}$ differs from $K_{1}$ as the participants must prove to know the contents of P without retransmitting $M_{1}$. Since the nodes are not reputed trustworthily, they have to prove that they know the plaintext P by producing a different ciphertext, i.e., $M_{2}$. Note that the increase in memory due to this second matrix key is irrelevant as the product nu bits reaches approximately 3 kB for each 100 house units. Note also that an external observer cannot learn how many offers are posed by the bidders as the sizes of $M_{1}$ and $M_{2}$ are always n×u. Moreover, $M_{1}$ and $M_{2}$ are cipher matrices that do not deliver any useful information for an external observer that does not know $K_{1}$ and $K_{2}$. Recall that, in step 1, along with the SM schedule, the TTP also informs in which second of the slot the node must confirm the data. Thus, step 4 consists of broadcasting $M_{2}$ to all nodes of the NAN during the second specified by the TTP, addressing the requirement of using time stamps to ensure the correctness of the sender identity.

### 4.2. Trading System Framework

**Figure 2:**
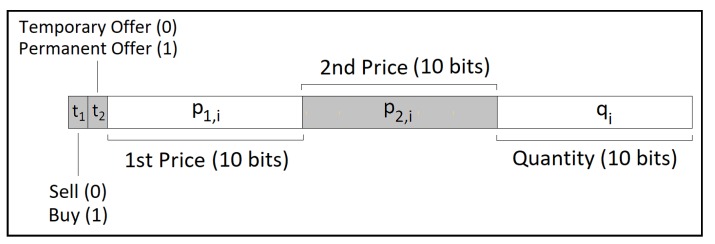
Bits of a bid string in terms of the bid content: The length of 32 bits comprises in this order $t_{1}$, $t_{2}$, $p_{1,i}$, $p_{2,i}$, and $q_{i}$.

The prices transmitted by each bidder are $P_{r,i}$ in

(8) $$P_{l}<P_{r,i}<P_{u},$$

where *r* indicates the round, with r∈{1,2} as the proposed system having two rounds, *i* denotes the node that submits the offer, and $P_{r,i}$ is the actual unitary price of the kWh offered by the *i*th node. Given that *v* out of the existing *n* SM owners forward offers, i∈{1,2,…,v}. The prices $P_{1,i}$ and $P_{2,i}$ are expressed in tenths of cents in order to reduce the probability of two offers having exactly the same bit sequence. Two bit strings $p_{1,i}$ and $p_{2,i}$ express the values of $P_{1,i}$ and $P_{2,i}$. Likewise, the quantity $Q_{i}$ of kWh in each offer is constrained to an interval $Q_{l}<Q_{i}<Q_{u}$, and thus, $Q_{i}$ is also denoted with an auxiliary bit string $q_{i}$. The bit strings $p_{1,i}$, $p_{2,i}$, and $q_{i}$ comprise 10 bits each. Instead of 1024 possible values, for simplicity, we reduce them to 999 values from 0000000001 up to 1111100111. Two bits complete the entire sequence, namely the type $t_{1}$ of the offer, with $t_{1}=0$ for sell and $t_{1}=1$ for purchase offers, and the status of the order in terms of time, with $t_{2}=0$ when the bid is valid only in the next trading session and $t_{2}=1$ to orders that stay valid throughout the day until a bid matches it. A length of 32 bits of the bid is complete with $t_{1}$, $t_{2}$, $p_{1,i}$, $p_{2,i}$, and $q_{i}$ gathered sequentially, as in Figure 2.

Purchasers must offer prices $P_{1,i}<P_{2,i}$, while sellers must set $P_{1,i}>P_{2,i}$. All prices of r=1 interact, producing deals as long as purchasers’ prices are equal or over sellers’ prices. Consequently, offers of r=2 are combined in order to achieve further deals.

When $t_{2}=1$, price uncertainty is eliminated for the other participants. This may cause several bids in the next time slot to have the same price values. In the case of this event, the time of arrival establishes the priority of all incoming bids. Therefore, for two bids with exactly the same prices, the one that arrives first at the TTP has the preference of the match.

A price mechanism is needed in order to enable the nodes to calculate the energy quantities and prices effectively traded from the knowledge of bit matrix P, as this calculation is an internal process of each node. As a way of providing such a mechanism for each node, our proposal consists of an adaptation of the Trade Reduction Mechanism (TRM) presented in [49], where the difference between specific sell and purchase offers, namely $b_{k}$ and $s_{k}$, yield a revenue to the auctioneer, which in our case means revenue for the utility company. Diversely, our proposal consists of producing revenues for the utility company at each deal, according to the prices and quantities negotiated. The numbers *m* and *n* denote the number of true purchasing and selling bids in P, of which the indexes are denoted by i∈{1,2,…,m} and j∈{1,2,…,n}, respectively. Purchasers must bid $P_{1,i}<P_{2,i}$, while sellers must follow $P_{1,j}>P_{2,j}$. The matches are developed first covering all possibilities with the first round prices and then with the second round ones. In the first round, only prices $P_{1,i}$ and $P_{1,j}$, for i∈{1,2,…,m} and j∈{1,2,…,n}, are taken into account, and after all possible first matches are computed, prices $P_{1,i}$ and $P_{1,j}$ are fully ignored and bid prices $P_{2,i}$ and $P_{2,j}$ are observed. Note that prices of both rounds do not communicate, and as such, the two rounds are completely independent. A second reason for this bid constitution is that, after computing bit matrix P, each node can differ the true offers from the false ones, as bit $t_{1}$ informs the relationship between both prices and computes all bids according to their prices and quantities without needing the support of the TTP to do so. Recall that P usually contains several false bids and each user that must identify them. For instance, if a given row of P presents $t_{1}=0$ and $P_{1,i}<P_{2,i}$, the node concludes that the corresponding row contains a false offer and thus ignores it.

The true offers of matrix P are reorganized in matrices $B\in Z^{4\times m}$ and in matrices $S\in Z^{4\times n}$, in which the columns are

(9) $$[\begin{array}{c} P_{1,i}\\ P_{2,i}\\ Q_{i}\\ k \end{array}],$$

i.e., the bids of the first and second rounds, the quantity offered,

(10) $$\left\{\begin{array}{c} k=i,\;\mathrm{for}\;\mathrm{matrix}\;B,\;\mathrm{and}\;\\ k=j,\;\mathrm{for}\;\mathrm{matrix}\;S. \end{array}\right.$$

Thus, *k* is the index of a purchasing or a selling bid according to the type of bid and the chronological criterion. In each round, the best purchasing and selling offers $P_{r,i}$ and $P_{r,j}$ are compared, and a deal is achieved as long as

(11) $$P_{r,i}\geq P_{r,j},$$

for i∈{1,2,…,m} and j∈{1,2,…,n}. In this case, the least quantity of kWh between the respective bids $Q_{i},Q_{j}$ is stored in $q^{\prime }$; the deal is carried out and the new best offers $P_{r,i}$ and $P_{r,j}$ are computed in a sequential process until Equation (11) no longer holds. Note that the PMD of [49] ignores the quantities of the bid in its matches. In the proposed framework, the financial reward to the auctioneer is given by

(12) $$U_{r}=\sum_{i,j}q^{\prime }\left(P_{r,i}-P_{r,j}\right),$$

where $P_{r,i},P_{r,j}$ are those of (11).

The system is described in Algorithm 1. The outcome of Algorithm 1 is credit assignments for each purchaser $B_{i}$, i∈{1,2,…,m} and seller $S_{j}$, j∈{1,2,…,n} as well as the aggregated revenue for the utility company given by Equation (12). We define a function f(X,r) that rearranges the columns of X so that the values of the *r*th row of X are sorted in accordance with Equations (1) and (2) given each case.
**Algorithm 1** Adapted Trade Reduction Mechanism (TRM) algorithm1:**procedure** Adapted TRM  (B,S)2:    $[\begin{array}{cccc} B_{1} & B_{2} & \ldots & B_{m} \end{array}]=\mathrm{zeros}\left(1,m\right)$3:    $[\begin{array}{cccc} S_{1} & S_{2} & \ldots & S_{n} \end{array}[=\mathrm{zeros}\left(1,n\right)$4:    **for** r=1:2 **do**5:        B←f(B,r)6:        S←f(S,r)7:        i←18:        j←19:        **while** B(r,i)≥S(r,j) **do**10:           $q^{\prime }\leftarrow \min\left\{B\left(3,i\right),S\left(3,j\right)\right\}$11:           $B_{i}=B_{i}+q^{\prime }B\left(r,i\right)$12:           $S_{j}=S_{j}+q^{\prime }S\left(r,j\right)$13:           $U_{r}\leftarrow U_{r}+q^{\prime }\left(B\left(r,i\right)-S\left(r,j\right)\right)$14:           $B\left(3,i\right)\leftarrow B\left(3,i\right)-q^{\prime }$15:           $S\left(3,j\right)\leftarrow S\left(3,j\right)-q^{\prime }$16:           **if** B(3,i)=0 **then**17:               $B\left(r,i\right)\leftarrow P_{l}$18:               i←i+119:           **end if**20:           **if** S(r,j)=0 **then**21:               $S\left(r,j\right)\leftarrow P_{u}$22:               j←j+123:           **end if**24:        **end while**25:    **end for**26:**return** $B_{1},B_{2},\ldots ,B_{m},S_{1},S_{2},\ldots ,S_{n},U_{r}$27:**end procedure**

Note that each node assembles B and S according to Equations (9) and (10) and performs the computations in Algorithm 1. Recall that each node is concerned only with the knowledge of all prices and quantities and, in case the node is a bidder, whether its own bid achieves a deal.

Since sellers and purchasers trade kWh over different quantities under time constraints and without disclosing any identities before making the offers public, our proposal is of a multidimensional, two-sided, sealed-bid, single-unit auction system.

### 4.3. Social Welfare of the Proposed System

A drawback of Equation (5) is that it does not take into account the welfare corresponding to the auctioneer itself, since it considers only the utility of purchasers and sellers. According to [49], it is important that the money earned by the mechanism be counted as part of the social welfare. In SG systems, it means that the reward due to the mechanism should be taken into account for social welfare calculation.

The trades between final prosumers substitute partially the energy supplied by the big seller, i.e., the utility company. As observed in Equation (5), the welfares $f_{i}$ and $g_{j}$ of purchasers and sellers are related to the difference of the actual clearing prices and to the willingness to pay or costs. Recall that captive consumers in a NAN are obliged to cope with prices $P_{l}$ and $P_{u}$ as shown in Equation (8). As a consequence, while prices $P_{l}$ and $P_{u}$ may not indicate the subjective expectations of local prosumers, they nevertheless inform the real prices that prosumers are obliged to practice in the case of not achieving any deal. In this sense, $P_{l}$ and $P_{u}$ give the virtual willingness to pay or costs referred to in the analysis of Equation (5) due to regulatory reasons.

**Figure 3:**
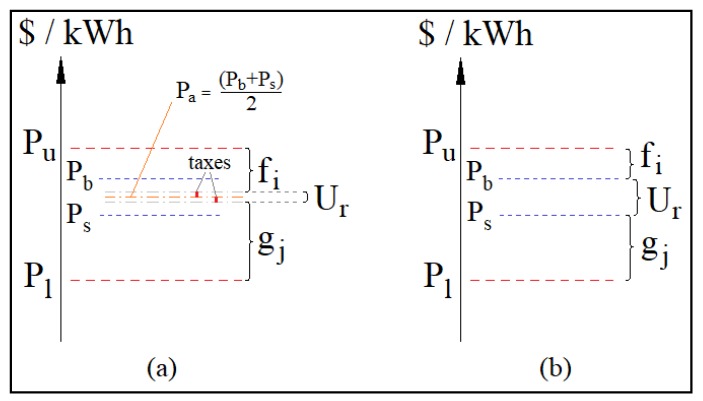
When the price of the purchaser equals or surpasses the price of the seller, $P_{b}\geq P_{s}$, (**a**) an average price $P_{a}$ is calculated in the state-of-the-art approaches of [35,36] and taxes are applied over the surpluses $\left(P_{b}-P_{a}\right)$ and $\left(P_{a}-P_{s}\right)$. (**b**) The proposed protocol, where the prices yield the welfares $f_{i}$ and $g_{j}$, which are complementary with regards to the welfare of the utility $U_{r}=P_{b}-P_{s}$, such that $f_{i}+g_{j}+U_{r}=P_{u}-P_{l}$.

Two fashions of accounting for revenues in a competitive framework are depicted in Figure 3. In both ways, it is assumed that $P_{b}\geq P_{s}$; otherwise, not a deal is achieved in a competitive system. In Figure 3a, the average price $P_{a}=\left(P_{b}+P_{s}\right)/2$ is the clearing price in [35,36]. Taxes are applied over the purchaser surplus $\left(P_{b}-P_{a}\right)$ and the seller surplus $\left(P_{a}-P_{s}\right)$. Such taxes may vary in terms of percentage, according to regulatory dispositions. We assign to the utility company the sum of both taxes as a way of rewarding it. Our proposal appears in Figure 3b, where we illustrate that, when there is a deal, the purchaser practices their own purchasing price $P_{b}$, the seller practices their price $P_{s}$, and the utility company receives the entire difference $\left(P_{b}-P_{s}\right)$. As such, our framework emulates the systems in [35,36] with taxes of 100%, i.e., the bidders cannot expect to pay less or to receive more than their original bids. Note that, in all cases, the welfares $f_{i}$ and $g_{j}$ are complementary to the welfare of the utility $U_{r}$ in such a way that $f_{i}+g_{j}+U_{r}=P_{u}-P_{l}$.

Note that, since the McAfee’s double-auction protocol does not consider quantities in each trade, in real applications with different quantities of a good under trade, sequential trades can be seen as several instantaneous McAfee’s deals, in which the average price $P_{a}$ stands for the price in Equation (3) at every interaction.

By including the utility company’s welfare in Equation (5), we have

(13) $$W\left(f,g,d\right)=\mathrm{Maximize}\;\sum_{i}f_{i}x_{i}+\sum_{j}g_{j}y_{j}+U_{r},$$

where $U_{r}$ is the revenue for the utility given by Equation (12). In practice, we rewrite Equations (5) as (13) by including the revenue of the utility company, as if the company was one seller or purchaser. Note that the component $\sum_{i,j}d_{i,j}z_{i,j}$ in Equation (5) stands for costs of the participants that are not computed in the general social welfare, constituting a case of welfare loss. In our framework, however, $\sum_{i,j}d_{i,j}z_{i,j}$ are the transaction costs which are fully included in $U_{r}$. In doing so, we achieve a full BB system. For more detailed information about the convergence of the trading system framework, we refer the reader to Appendix A.

## 5. Results

In Section 5.1, we illustrate the performance of our framework in terms of security requirements, and in Section 5.2, we undertake a comparison with the state-of-the-art privacy and trading systems.

### 5.1. Security and Computational Cost Analysis

#### 5.1.1. Privacy Threats Along Stages of the Proposed Framework

The threat model establishes that all nodes are malicious. In the setup zero stage defined in Section 4, attackers seek access to each SM unique parameter provided by the TTP-authorized vendor. Their aim is to impersonate an authenticated node inside the network in a attempt to learn the identities of the bidders. Moreover, malicious users may induce all other nodes in error by broadcasting modified data in an attempt to gain financial advantage. Additionally, they may access all links between the TTP and final nodes in order to disrupt the grid by harming broadcasted content.

In the D-Y adversary model, an attacker is assumed unable to break cryptographic primitives [9]. Thus, the slot zero and the bids submission stage are considered data leakage proof as the required time for breaking AES keys is in the order of $10^{37}$ seconds [69]. Note that a node that forwards an offer in the bids submission stage includes its own IP in the plaintext, which is encrypted, and that the TTP confirms the arrival of any valid offer to its author. Furthermore, the exact instant of this message forward is uncertain over the entire duration of the time slot. A successful attack in this case demands continuous and explicitly intrusive actions. This collides with the notion in [75], according to which malicious data attacker are supposed to compromise as few data as possible in order to inject undetectable attacks with the lowest cost and effort. Hence, the probability of successful attacks in the slot zero and bids submission stage is assumed significantly unlikely.

**Figure 4:**
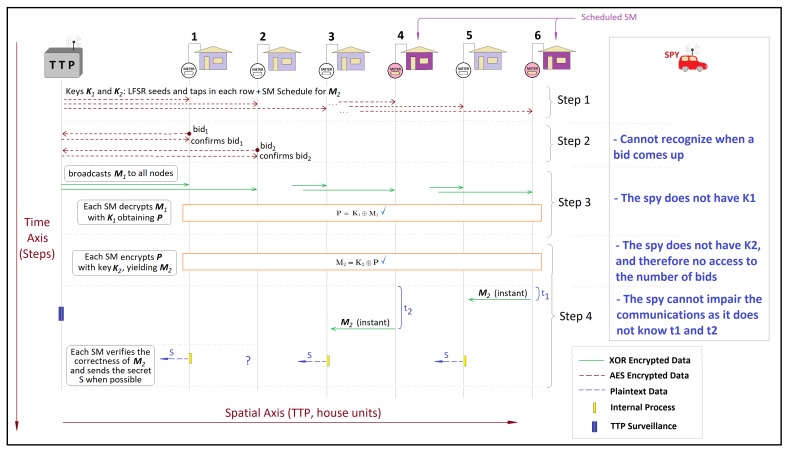
Sequence of stages in the proposed framework from the standpoint of a spy: Due to the sequence of stages, the spy can neither infer how many orders are posed nor of which type they are.

In terms of data protection, it is useful to see that, for an external observer, the messages from the SMs and the TTP can be of any content as all nodes are supposed to exchange data with the TTP informing for instance the node availability, voltage measurements, etc. In Figure 4, the adversary is represented by the red vehicle. It receives also dummy packets that can be exchanged between TTP and the SMs in order to thwart traffic analysis attacks. As a consequence, it is not possible for the adversary (spy) to infer the purpose of such messages. For internal attackers, i.e., those who possess an SM and are authenticated, it is equally not possible to devise the contents of the messages exchanged by the TTP and the other nodes, given that all the AES keys are different. As a consequence, in the slot zero and in the bids submission stages, the external observer can infer no useful information. In the bids disclosure stage, the bit matrix $M_{1}$ is broadcast to all SMs of the NAN. The nodes are now more vulnerable to attacks on the data content since the adversary can see the TTP broadcasting data of nu bits, which is always the dimension of $M_{1}$. The spy might decide to try to alter the ciphertext bits deliberately; however, in order to compromise the entirety of broadcast data, the attacker must access all links between the TTP and final nodes, a very problematic task if the network is sufficiently spread spatially. In the bids verification stage, the matrix $M_{2}$ has, in comparison to the bit matrix $M_{1}$, two additional protections: specified nodes are programmed to broadcast it and in specific time windows—in Figure 4, house units 6 and 4 are the scheduled nodes and have the time windows t1 and t2, respectively—as a case of time-stamp application. Only when the attacker knows in advance which nodes are scheduled to broadcast $M_{2}$ in the respective time slot can it harm the broadcasts content. However, it is taken as impossible due to the lack of access to the AES keys between the TTP and the other nodes, which derives from the D-Y adversary model. Note that, due to the sequence of stages, the spy can neither infer how many orders are posed nor of which type they are.

Suppose that the attacker is an internal node and that it is scheduled to broadcast $M_{2}$. It can broadcast a different matrix, say $M_{2}^{\prime }$, to try to induce errors in all other nodes. However, such an attack cannot avoid the nodes receiving the correct $M_{2}$ from other scheduled nodes. Moreover, the TTP undertakes strict surveillance over the broadcasted $M_{2}$ contents. A node that broadcasts a false version $M_{2}^{\prime }$ is included in the Revocation List, even when it transmits the correct share $E_{j}$. The protocol admits that a scheduled node does not broadcast $M_{2}$, since it might not have received $M_{1}$ due to package losses. In comparison to an external observer, which is not authenticated, an internal node can learn the number and the types of bids; however they are not able to link them with the respective authors.

#### 5.1.2. Security and Privacy Features

We classify our framework under the requirements of anonymity, untraceability, no impersonation, unforgeability, non-repudiation, verifiability, non-linkability, linkability within a single bidding round, privacy, forward security, authenticity, and integrity as presented in [76].

Anonymity is achieved when no unauthorized entity is able to identify the bidder during the bidding. Our system accomplishes this goal via AES keys between each bidder and the TTP. Dummy packets and the constant size of matrices $M_{1}$ and $M_{2}$ avoid identification by means of traffic analysis. Untraceability is attained when the bid winner cannot be identified at the end of the bidding by untrusted entities. However, the winning bidder’s legitimacy should be verifiable. Furthermore, no individual should be traceable during a bidding round. Our framework meets this requirement as the matrices $M_{1}$ and $M_{2}$ deliver and confirm all informations about the bids; all nodes can know the winner bid. Note that the winner identity is never accessible for any node.

When no one participates in the bidding with the identity of another bidder, no impersonation is achieved. Since all nodes are only admitted when their ciphertexts include their IP into the AES encrypted message, the TTP cannot accept false participants. Unforgeability is fulfilled when no one is able to falsify a valid bidding price. In the proposed framework, it derives from the no impersonation requirement.

Non-repudiation is observed when the bidders cannot deny their bid after the winning bidder has been announced. After sending a bid and after the TTP accepts, the node cannot deny its offer, which makes part of matrices $M_{1}$ and $M_{2}$, as the TTP is assumed inviolable. Verifiability is achieved when anyone can verify the validity of the bids. This relies on the fact that matrix $M_{2}$ reproduces the content of matrix P from what is informed by $M_{1}$.

Non-linkability among various bidding rounds consists of no participant being able to access results that enable a bidder to be identified in various bidding rounds. Due to the invariable sizes of $M_{1}$ and $M_{2}$, it is impossible even for authenticated nodes to know when a node submits bids. Linkability within a single bidding round is achieved when anyone can determine the number of times a bidder has bid, which in the case of the proposed framework is straightforward as it imposes that each node bids a maximum of only once per time slot. In terms of privacy, untrusted entities must not be able to link bids to individual consumers. Moreover, they must not be able to infer private information about individual consumers. It is applicable even for internal nodes.

Even if the current bidding key is compromised, no information about the previous keys should be leaked, which is the concept of Forward Security. The LFSR ensures that the XOR keys are not repeated from one time slot to another, making it infeasible to access the previously submitted bids. Authenticity and integrity of all bid notifications occurs when all bids are be verifiable. The TTP verifies it with regards to each ciphertext received.

The single registration requirement consists of a bidder being required to register in the system only once, and then, they can participate in all future bid sessions. In our framework, this is provided with the secrecy of the XOR keys with their seeds and taps. Using matrices $K_{1}$ and $K_{2}$, every node can forward intelligible bids, when it is automatically admitted as a participant. Easy revocation is defined as the ease for the registration manager to revoke a bidder. In case of errors, the TTP in our system easily revocates a scheduled node that broadcasts false contents or a bidder that tries to submit an impossible bid—for instance, a purchasing bid in a household that has not generate assets. Incentive allocation consists of the bid winner being able to claim the incentive without revealing their identity, and no other entity should be able to impersonate the winner. The winner can claim the incentive by messages between it and the TTP. Furthermore, in case of the same bid prices, the TTP chooses the winning bid with a temporal criterion, i.e., that is forwarded first and can inform this fact to a bidder that claims a deal.

### 5.2. Comparison with the State-of-the-Art Privacy and Trading Systems

Comparing the proposed framework with the state-of-the-art ones, Table 2 shows the results in terms of privacy in the SG, with 1 for existing and 0 for nonexisting features. As a privacy enhancement methods [1,67,76] use DLP-based encryption systems, which have a comparatively high computational complexity. In [77], nodes embedded with distributed controllers coordinate with neighboring peers in order to find the optimal operating data, such as instantaneous consumption power. In doing so, they transmit plaintext information, and therefore, none of the common cryptographic techniques is applied. In [11], employing a large number of anonymous addresses is part of the solution for privacy attainment, contrary to our framework and to the discussion of Section 2.3. The address of each SM is disclosed in [76] and in the proposed framework only to a central controller, while in [77], it is disclosed only to one-hop nodes. Interested parties can possibly be identified as such by a malicious observer in all compared systems by means of traffic analysis or address de-anonymization, i.e., an observer can identify the role of a participant by such attacks, while in our system, it is completely avoided. The proposed framework is de-anonymization proof since, even when the IP of a node is identified, an observer cannot conclude if the node is a bidder. For instance, note that the SM schedule establishes that nodes that do not take part in the bids forward the matrix $M_{2}$.

In Table 3, the proposed framework and the state-of-the-art schemes are compared in terms of pricing systems. Our framework allows for free-price formation and a variety of different auction systems, as for instance the suggested one [49]. The proposed framework also dismisses previous information about energy consumption profile. Such data are a requisite for the systems in [41], which characterizes the operation for the benchmark scenario of a DR market where the operator has full information of all DR-related parameters, such as the utility function of the consumers, which is representative of their consumption profile and decision-making process. The proposed framework also allows the inclusion of storage elements, which are excluded from the systems such as in [20], which develops an energy-trading system of a community energy storage (CES) device for demand-side load management within a neighborhood area network. The energy users that have their own photovoltaic power generation are allowed to trade energy from their personal surplus with the grid and the CES device in a competitive game framework. Pricing freedom is fully guaranteed in [3,34]. In [53], a power market scheduling center (PMSC) is proposed, which manages all the energy providers and makes them provide a unified price to the subscribers, and energy providers generate the optimal quantity of electricity to get maximum utilities. In [41], aggregators provide DR services to the operator and guarantee a reduced electricity bill to the end users, negotiating with both sides in order to maximize its own profit. In [24], a regulatory authority calculates the instantaneous prices that minimize the total social cost based on the knowledge of the utility functions of the associated consumers, establishing a unique value to them. In these examples, the final consumers are limited to playing the role of price takers.

## 6. Conclusions

As an emerging cyber-physical system, the SG is attractive for enabling distributed energy control, allowing for high levels of automation and security of the power system. Power is already produced inside the boundaries of final user real states and is exported to the company or to other consumers. As a consequence, the old grid structures must be reformulated. With regards specifically to the prices in the new electrical systems, a key aspect is that the utility company usually sells the energy for a unitary price $P_{u}$ to their consumers and buys back the energy of prosumers for a different unitary price, $P_{l}$, yielding the range for dealing prices between final users.

In the proposed framework, we provide an effective approach for privacy protection for prosumers in a NAN that takes into account the problem of self-interested players that intend to trade energy in the context of a NAN. We additionally present a clear pricing mechanism that allows for each smart meter to calculate the final prices and respective amounts of energy that are traded, including a financial reward to the power-grid company, while keeping all identities secret. The communication links are provided by a patent-pending proprietary communication system. Our results show higher consistency when compared to the state-of-the-art models, especially in what concerns privacy protection against IP de-anonymization and traffic analysis attack. In order to achieve these objectives, we use AES 128 bit associated with LFSR-based XOR matrices, which have constant sizes, independent of the number of bidders. In doing so, our cryptographic framework has a considerable low computational cost.

Concerning future works, a first step is to monitor the trends of the SM industry in order to determine whether off-the-shelf SM can utilize asymmetric keys and can resist active attacks of all types. Tests with real SMs in a NAN are also required in order to measure typical values of package losses and Signal no Noise Ratio (SNR) as a function of the NAN infrastructure and topology. Finally, the proposed energy-trade model must undergo regulatory discussions before it can be implemented in realistic scenarios.

## Figures and Tables

**Table 1:** Commercial treatment of energy and the respective approaches in the literature.

Criterion	Cooperative Games	Competitive Games
How energy is predominantly seen	as a public good	as a commodity
Main goals	social fairness, market control	decentralization, market efficiency
Main references	[17,18,22,23,24,25,26,27,28,29,30,31,32,33]	[3,4,19,20,21,34,35,36,37,38,39,40,41,42]

**Table 2:** Comparison between the proposed framework and the state-of-the-art approaches in terms of privacy.

Reference	[1]	[67]	[76]	[77]	[11]	Proposed
Privacy enhancement method	DLP-based public key + secure hash algorithm	DLP-based public key (Boneh- Goh-Nissim)	DLP-based public key (ElGamal)	None	Anonymous Addresses + cryptographic signature	AES keys + XOR matrix of fixed length
Cost of cryptography	High	High	High	None	Medium	Low
Disclosure of SM address	1	1	1	1	0	1
Dismissal of secure communication channel	1	1	0	0	1	1
ID de-anonymization proof	0	0	1	0	0	1
Absence of need for connection between each pair of nodes	0	1	1	1	0	1
Impossibility of interested parties identification	0	0	0	0	0	1

**Table 3:** Comparison with the state-of-the-art approaches in terms of pricing systems.

Reference	[53]	[33]	[41]	[78]	[18]	[22]	[20]	[23]	[24]	[3]	[16]	[34]	Proposed
Dismissal of consumption profile assumptions	0	0	0	0	0	1	0	0	1	1	1	1	1
Possibility of storage elements inclusion	0	1	0	1	0	1	0	1	0	0	1	1	1
Pricing freedom	0	0	0	0	0	0	0	0	0	1	0	1	1
Inclusion of a TTP	1	1	1	0	1	1	0	1	1	1	1	0	1
Competitive market	0	0	1	1	1	0	1	0	0	1	0	1	1

## Abbreviations

The following abbreviations are used in this paper:

NAN	Neighborhood Area Network
AES	Advanced Encryption Standard
XOR	Exclusive-OR
SGs	Smart Grids
SM	Smart Meters
HAN	Home Area Network
DER	Distributed Energy Resources
NEM	Net Energy Metering
KWh	kilowatt-hours
AMS	Advanced Metering Structure
IP	Internet Protocol
WAN	Wide Area Network
DLP	Discrete Logarithm Problem
DSO	Distribution System Operator
TTP	Trusted Third Party
LFSR	Linear-Feedback Shift Register
SNIR	Signal-to-Noise-plus-Interference Ratio
PSK	Phase Shift Keying
QPSK	Quadrature Phase Shift Keying
CSS	Chirp Spread Spectrum
BER	Bit Error Rate
TRM	Trade Reduction Mechanism
PMSC	Power Market Scheduling Center

## Appendix A

This appendix presents an example conceived to clarify the convergence of the trading system framework discussed in Section 4.1. **Example 1**. Let a NAN be 150 house units numbered from 1 to 150, from which 110 are equipped with an SM. Among all 110 authorized nodes, 4 purchasers and 4 sellers forward their bids in a given time slot of the day, such that n=110 and v=8. After recognizing the origin and validity of each bid in accordance to their AES key as explained in Section 4.1, the TTP knows all offers and sets up the content in Table A1. In this example, *H* informs the number of each participating house unit, the pair $t_{1}t_{2}$ are directly given by bits, while the prices $P_{1,i}$ and $P_{2,i}$ and the amount of energy $Q_{i}$ are informed by their values after conversion. Furthermore, let $P_{l}=1500$ and $P_{u}=2000$, so that the range of $P_{r,i}$ as given by Equation (8) is between $1.50/kWh and $2.00/kWh.

**Table A1:** Bids as received by the TTP in a given time slot—Example 1.

Purchasers					Sellers				
H	$t_{1}t_{2}$	$P_{1,i}$	$P_{2,i}$	$Q_{i}$	H	$t_{1}t_{2}$	$P_{1,i}$	$P_{2,i}$	$Q_{i}$
80	11	1555	1556	80	31	00	1800	1790	140
3	10	1820	1830	90	142	00	1900	1870	200
115	10	1501	1877	100	99	00	1910	1920	65
77	10	1845	1846	30	10	00	1960	1580	70

From all the bids of Table A1, note that the bid of house unit 99 is inconsistent, as sellers must set $P_{1,i}>P_{2,i}$. As a consequence, the TTP does not take this offer into account when assembling the matrix P that is sent to all nodes by means of $M_{1}$. Furthermore, the node 99 is included by the TTP in the Revocation List, staying unable to take part in future bids until the problem with the node is clarified and fixed. Note also that, while assembling the matrix P, the TTP removes the information about columns *H* shown in Table A1. Hence, all nodes receive only the data related to the bids and not to their authors, which are learned only by the TTP.

By applying Algorithm 1, two rounds are performed: first with r=1 and only then with r=2. In summary, at each step, the lowest seller and higher purchaser prices are combined until the lowest energy quantity between them is achieved. Starting with r=1, only the prices $P_{1,i}$ are considered. Observing Table A1, the best offers from each side purchase 30 kWh at $1.845/kWh and sell 140 kWh at $1.80/kWh from house units 77 and 31, respectively. The lowest energy amount, i.e., 30 kWh, is the quantity that is effectively traded between them. The prices of each side are practiced, so that the utility company receives the difference. Hence, at this first step, house unit 77 spends $1.845/kWh for buying 30 kWh from house unit 31, which receives $1.80/kWh for the 30 kWh, while the utility company is rewarded with 30 × $0.045 = $1.35.

As house unit 31 has still 110 kWh available after supplying house 77, the next best purchaser is considered—if its offer is compatible with the seller value, a new deal is achieved. Still with r=1, house unit 3 intends to purchase 90 kWh at $1.82/kWh. Since it offers a higher price than the seller, house 3 obtains 90 kWh from house 31, paying 90 × $1.82 = $163.8, while house 31 receives 90 × $1.80 = $162. The difference of $1.8 is appropriated by the utility company.

At this point, house unit 31 still has 20 kWh to trade. However, the best remaining purchase offer is lower than the selling price. Now, all deals with r=1 are finished and the second round, r=2, is started.

The best prices $P_{2,i}$ from the purchase side is $1.877/kWh for 100 kWh from house unit 115. It covers the sellers’ offer of 70 kWh from house 10, the remaining 20 kWh from house 31, and part of the offer from house 142. After these deals, house 142 has still 190 kWh to sell at $1.87/kWh. However, the best remaining $P_{2,i}$ from the purchase side is now $1.846/kWh. At this point, all the deals are finished for this slot.

Note that, in this example, the offer from house unit 80 is not considered in any deal. However, it is the only bid with $t_{2}=1$, making it valid throughout the day until a match is obtained. Since all the bidders learn that a purchase offer of $1.555/kWh is valid for the next slot, the bids at this price that arrives at the TTP are considered to have the preference in accordance to the instant of the bid arrival.

All the routines of this example are performed internally by each node from the knowledge of matrix P. Therefore, by the end of the Algorithm 1 application, all nodes learn which quantities were traded at each prices; however, not knowing the identities of the bidders.
